# Malaria transmission in Nepal under climate change: anticipated shifts in extent and season, and comparison with risk definitions for intervention

**DOI:** 10.1186/s12936-022-04417-x

**Published:** 2022-12-22

**Authors:** Shreejana Bhattarai, Jason K. Blackburn, Sadie J. Ryan

**Affiliations:** 1 Quantitative Disease Ecology and Conservation (QDEC) Lab, Department of Geography, University of Florida, Gainesville, FL USA; 2 Emerging Pathogens Institute, University of Florida, Gainesville, FL USA; 3 Spatial Epidemiology and Ecology Research (SEER) Laboratory, Department of Geography, University of Florida, Gainesville, FL USA

**Keywords:** *Anopheles stephensi*, Nepal, Climate change, Elimination, Intervention

## Abstract

**Background:**

Climate and climate change affect the spatial pattern and seasonality of malaria risk. Season lengths and spatial extents of mapped current and future malaria transmission suitability predictions for Nepal were assessed for a combination of malaria vector and parasites: *Anopheles stephensi* and *Plasmodium falciparum* (ASPF) and *An. stephensi* and *Plasmodium vivax* (ASPV) and compared with observed estimates of malaria risk in Nepal.

**Methods:**

Thermal bounds of malaria transmission suitability for baseline (1960–1990) and future climate projections (RCP 4.5 and RCP 8.5 in 2030 and 2050) were extracted from global climate models and mapped for Nepal. Season length and spatial extent of suitability between baseline and future climate scenarios for ASPF and ASPV were compared using the Warren’s I metric. Official 2010 DoHS risk districts (DRDs) and 2021 DoHS risk wards (DRWs), and spatiotemporal incidence trend clusters (ITCs) were overlaid on suitability season length and extent maps to assess agreement, and potential mismatches.

**Results:**

Shifts in season length and extent of malaria transmission suitability in Nepal are anticipated under both RCP 4.5 and RCP 8.5 scenarios in 2030 and 2050, compared to baseline climate. The changes are broadly consistent across both future climate scenarios for ASPF and ASPV. There will be emergence of suitability and increasing length of season for both ASPF and ASPV and decreasing length of season for ASPV by 2050. The emergence of suitability will occur in low and no-risk DRDs and outside of high and moderate-risk DRWs, season length increase will occur across all DRD categories, and outside of high and moderate-risk DRWs. The high and moderate risk DRWs of 2021 fall into ITCs with decreasing trend.

**Conclusions:**

The study identified areas of Nepal where malaria transmission suitability will emerge, disappear, increase, and decrease in the future. However, most of these areas are anticipated outside of the government’s current and previously designated high and moderate-risk areas, and thus outside the focus of vector control interventions. Public health officials could use these anticipated changing areas of malaria risk to inform vector control interventions for eliminating malaria from the country, and to prevent malaria resurgence.

**Supplementary Information:**

The online version contains supplementary material available at 10.1186/s12936-022-04417-x.

## Background

Malaria mapping has a lengthy history, and malaria maps have long been used for public health reference and planning, and for global malaria control and intervention efforts [1]. The recent increase in availability of large-scale disease and vector surveillance data sets has led to a surge in malaria mapping projects and publications [2–10]. Two main approaches to mapping malaria risk can be broadly described as: (1) spatial data modelling—applying statistical models to geospatially explicit epidemiological or surveillance data sets, such as empirical Bayesian estimation [11] or ecological niche modelling [6, 12]; or (2); creating mechanistic models of the transmission process and projecting those onto landscapes [5, 7, 8, 13–16]. The latter approach includes temperature-dependent prediction models, incorporating non-linear temperature dependent mosquito and parasite development traits into transmission models [5, 7, 8, 15, 17], which can then be used to map the potential for temperature-driven malaria transmission suitability risk, and the shifting risk of malaria with climate change. Importantly, temperature-driven mechanistic transmission models allow the projection of malaria transmission suitability risk onto landscapes, despite the presence of control measures, which may affect reported data used for statistical epidemiological estimation in spatial data models.

Climate change affects the spatial pattern of malaria risk because malaria transmission occurs in areas with suitable climate for *Anopheles* spp. mosquitoes and *Plasmodium* spp. parasite development. Thus, as the climate changes, geographic shifts in malaria transmission and changes in the length of the transmission season will occur [8]. Previous studies have shown that there have been shifts in spatial patterns of malaria due to climate change [18, 19] and several studies have projected the shifts in potential malaria transmission patterns in the future with climate change in various parts of the world [7, 8, 20–22]. In Nepal, climate change has been shown to have already affected malaria transmission [23–26]; a 1 °C increase in minimum temperature was linked to a 27% increased malaria incidence in a study in Dhangadi and Morang districts [25], and a study in the Jhapa district found that malaria cases increased with minimum increase in temperature and increased with a considerable decrease in total rainfall [24]. Additionally, malaria hotspots shifted within Morang district [25], and another study found that malaria incidence increased in recent years particularly in the hills and mountains of Nepal, indicating that malaria is shifting to new locations previously considered malaria-free [23]. No studies have thus far explored the potential future climate impact on malaria transmission in Nepal, a point emphasized in a recent malaria stratification report of 2018 [27].

In this study, baseline and future climate-driven malaria transmission suitability risk in Nepal were mapped and contrasted with district-level risk designations (DRDs) reported by Department of Health Services (DoHS) in 2010, and with the sub-district malaria risk wards (DRWs) designated in 2021 for ongoing elimination campaigns. These mapped risks were also contrasted with incidence trend clusters (ITCs) based on district level incidence trajectories spanning 2005–2018 [28]. To describe baseline and potential future malaria transmission suitability in Nepal, published models of thermal transmission suitability [5, 29] was used to map suitability as a function of temperature across the landscape. For this study, the transmission models for thermal suitability for a combination of *Anopheles stephensi* and *Plasmodium falciparum* (ASPF) and *An. stephensi* and *Plasmodium vivax (ASPV)* from Villena et al. [29] were used. Specifically, temperature dependent mechanistic models were used to assess suitability of malaria transmission by *An. stephensi* at baseline climate conditions, and in the future in Nepal in 2030 and 2050. Describing climate-induced suitability changes from a baseline (1960–1990), prior to the start of intervention measures, to the nearer term 2030, and onward to 2050, presented a backdrop to both the ITC analysis and to the DoHS designated risk areas at the district level (2010) and more fine-scaled ward-level risk designations (2021).

*Anopheles stephensi* is a major malaria vector in southern and western Asia including in Nepal’s neighboring country of India [12], with documented presence in the mountainous Garwhal region, lying just to the Northwest of Nepal (near Nanda Devi), with reported observations ranging between 300 and 800 m in altitude [30]. It is a competent malaria vector adapted to the thermal regimes of this region, and thus is likely representative of malaria transmission conditions in Nepal, despite not currently addressed as a focus of malaria efforts. It is currently undergoing expansion from its native range(s), emerging, and establishing in new countries and even novel regions [29]. The dominant reported malaria vectors in Nepal are *Anopheles fluviatilis*, *Anopheles annularis* and *Anopheles maculatus* [27, 31, 32]. Unfortunately, thermal traits data are not available to fully parameterize malaria thermal transmission suitability models for these vectors. *Anopheles stephensi* is the Asian malaria vector which has the most laboratory derived thermal traits data available for calculating the malaria transmission suitability metric [29]. Thus, *An. stephensi* is used both as a proxy for existing dominant malaria vectors in Nepal, and as a potential expanding malaria vector.

### Previous descriptions of malaria risk in Nepal

Malaria risk stratification in Nepal was conducted at the district level by the DoHS in 2010, using reported malaria incidence, and the population of the district was defined as the population at risk of malaria [33]. In this study, these were referred as *DoHS risk districts*, abbreviated to ‘DRDs’. With the substantial decline in malaria burden over the past decade or so, and the evidence that only few areas within the district reported malaria cases while other areas remained free of malaria, the first sub-district level malaria risk stratification and designations were established at the Village Development Committee (VDC) level in 2013 [27]. Following that, with further decline in malaria in the country, and the recognition that malaria risk is not homogenous within a VDC, malaria risk stratification was made at an even finer scale, wards, and called microstratification. In 2016, malaria risk stratification was conducted at the ward level [31], and since 2018, it is conducted annually. Ward level stratification designates high-risk wards, moderate-risk wards, low-risk wards, and no-risk wards. In this study, these were referred as *DoHS risk wards*, abbreviated to ‘DRWs’. This ward-level stratification was based on three factors that determine malaria transmission: disease burden (API-confirmed malaria cases per 1000 risk population) in the previous three years; suitability characteristics of the area such as climate, ecology, and the presence or abundance of key vector species; and the potential vulnerability of the area to malaria (in terms of human population movement) [27]. This process of risk stratification was undertaken to prioritize vector control interventions in high-risk areas and moderate-risk areas. In Nepal, two major vector control intervention strategies are used. Long-lasting insecticidal nets (LLINs) are mass distributed in high and moderate-risk areas, and in regular intervals, to people living in the active foci to ensure universal coverage, and to pregnant women during antenatal care visits to health care institutes in high and moderate-risk areas, and to mobile and migrant populations [34]. Indoor residual spraying (IRS) is conducted twice a year in selected districts, and twice a year, focal spraying is conducted to eliminate high risk foci (wards) [34].

To assess the progress and impact of vector control interventions in Nepal, in a previous study, we evaluated whether malaria case rates reported between 2005 and 2018 were increasing or decreasing and identified clusters of districts where that occurred at a rate different than the districts not in the clusters [28]. In this study, these areas are defined as *incidence trend clusters*, abbreviated to ‘ITCs’*.* While there was an overall declining trend of malaria in Nepal during the period, several clusters of districts were identified with high or low temporal trends, for five malaria case indicators (Indigenous malaria, Imported malaria, PF malaria, PV malaria, and Total malaria). In that study, it was found that in addition to other malaria indicators, PF and PV malaria also have several clusters of high temporal trends which means that PF and PV malaria are increasing in some clusters in the country. The increasing trend of malaria is not encouraging for a country which is aiming to eliminate malaria by 2026, and which is implementing vector control interventions like LLINs and IRS. Clusters with increasing trends occurred not only in southern districts where malaria is historically endemic but also in high altitude northern districts where malaria is not endemic.

Climate change may facilitate the establishment of malaria at high altitudes. As previous studies have shown climate change is already affecting malaria transmission in Nepal, it was important to understand how climate change may affect malaria transmission suitability in the future, and impact the country’s planned goal, to eliminate malaria. Thus, the objective of this study was to map and describe the predicted baseline and future geographic distribution of malaria transmission suitability in Nepal and compare these estimates with observed data for DRDs, DRWs and ITCs. Nepal is preparing for malaria elimination by 2026 and these results can be valuable for planning efforts to eliminate malaria and prevent malaria resurgence.

## Methods

### Climate data

To examine the impact of climate change on transmission risk, the approach of Ryan et al. [35] was followed. A global ‘baseline’ climate data set was obtained at a 5-arc minute resolution, the WorldClim baseline data (WorldClim version 1.4 /http://worldclim.org). This climate data is based on monthly means for the period 1960–1990. This 30 year reference period is usually employed to represent the baseline conditions from which future changes are estimated. To capture a changing climate, near-term future projections in 2030 (2021–2040 mean) and 2050 (2041–2060 mean) were used. Future scenario climate model output data were acquired from the research program on Climate Change, Agriculture, and Food Security (CCAFS) web portal (http://ccafs-climate.org/data_spatial_downscaling/), part of the Consultative Group for International Agricultural Research (CGIAR). Calibrated model outputs created using the delta downscaling method, from the IPCC AR5 were used. For the future projections, four General Circulation Models (GCMs): Beijing Climate Center Climate System Model (BCC-CSM1.1); the Hadley Centre Global Environment Model version 2, HADGEM2-AO and HADGEM2-ES); and the National Center for Atmospheric Research’s Community Climate System Model (CCSM4) [35] were used under two greenhouse gas emission scenarios: RCP 4.5 and RCP 8.5. The projected data here consisted of the ensemble mean of the 4 GCMs. The datasets were obtained at a resolution of 5-arc min matching the baseline data.

### DoHS risk districts (DRDs)

**Fig. 1:**
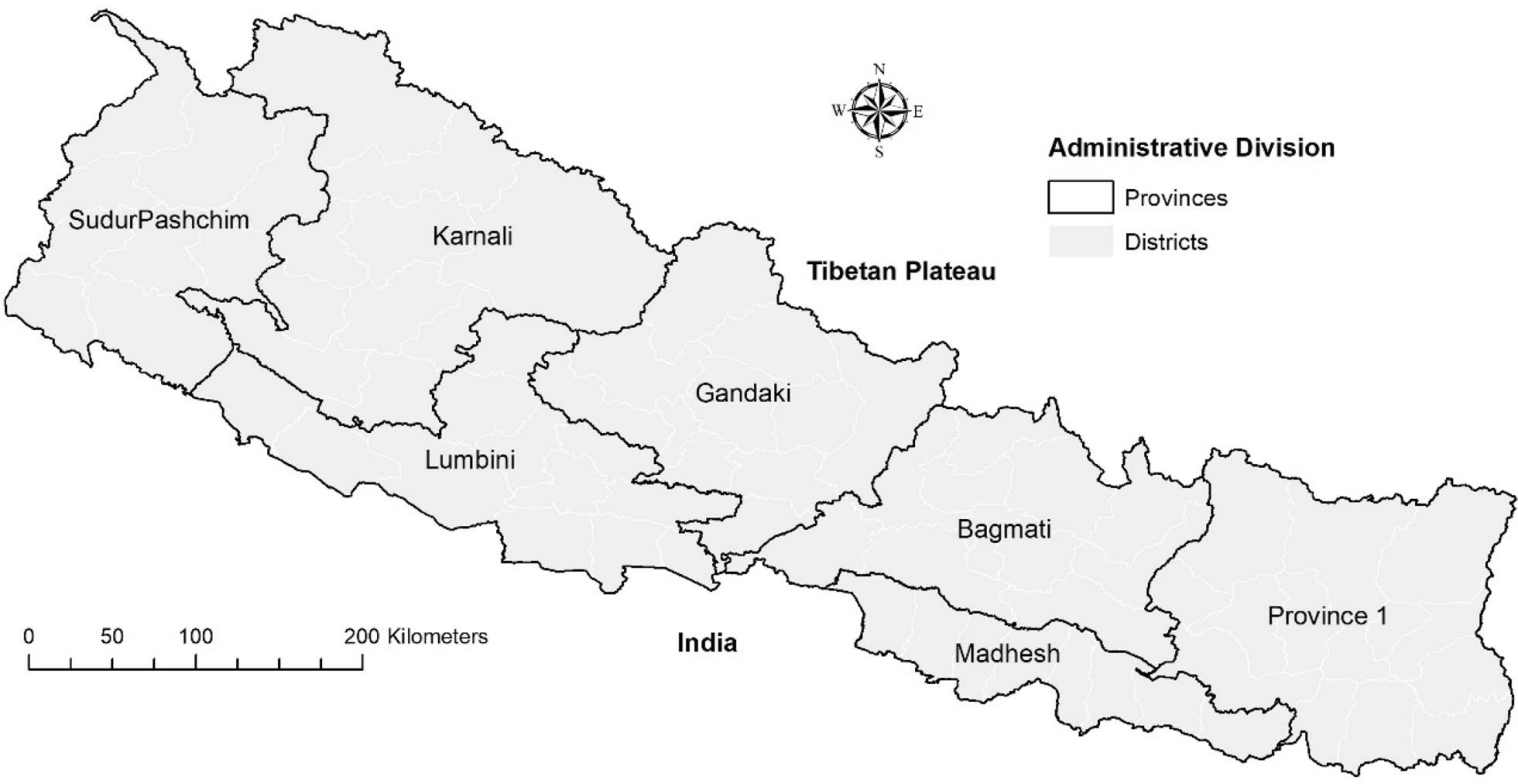
Administrative divisions of Nepal. There are 7 provinces and 77 districts in Nepal. Province 1 has yet to receive its name

In 2010, the DoHS conducted malaria risk stratification in Nepal at the district level based on Annual Parasite Index for implementing vector control interventions. According to the stratification, there were high-risk, moderate-risk, low-risk, and no-risk districts. Using a shapefile of seventy-five districts (2 of which divided to later become 77 districts (Fig. 1)), the four categories of malaria risk were assigned to the districts and created a shapefile in ArcGIS 10.6.1 (ESRI, Redland, CA).

### DoHS risk wards (DRWs)

In 2016, the DoHS initiated malaria risk microstratification at the wards level (sub-district) based on three key determinants: disease burden, receptivity characteristics, and vulnerability, for implementing vector control interventions. The microstratification has classified wards of Nepal into four categories: high-risk, moderate-risk, low-risk, and no-risk wards. Although the stratification has categorized four categories of malaria risk, the data is available only for high-risk and moderate-risk wards. The details of methodology of the microstratification are described elsewhere [27, 31]. This ward level microstratification has been conducted every year since 2018. For this study, the most recent microstratification categories of 2021 were used. Using a shapefile of the wards in Nepal, the two categories of malaria risk were assigned to the wards: high-risk, and moderate-risk wards and a shapefile was created in ArcGIS 10.6.1 (ESRI, Redland, CA).

### Incidence trend clusters (ITCs)

In a previous study, incidence trend clusters (ITCs) of PF and PV malaria were identified by using Spatial Variation in Temporal Trend (SVTT) method in SaTScan [36]. Briefly, SVTT identifies the most likely spatial cluster and compares the rate of change within the cluster to the rate of change outside of the cluster [36]. ITCs were mapped at the district level with blue colors indicating decreasing rates of malaria within the cluster compared to outside. Districts with increasing rates were colored in red. All primary and secondary clusters were considered so long as they were significant at the 5% significance level. Further details about the data, methods, and our approach are explained elsewhere [28]. In this study, ITCs relative to DRDs as defined in 2010, DRWs as defined in 2021, and to the modelled climate driven transmission suitability were examined.

### Malaria transmission suitability model

Temperature-dependent malaria transmission suitability were mapped for Nepal using recently published experimentally derived thermal suitability models for malaria transmission by *An. stephensi*. The models assess temperature-dependent malaria transmission suitability by two mosquito-parasite combinations: *An. stephensi* and *P. falciparum* (ASPF) and *An. stephensi* and *P. vivax* (ASPV) [29]. Briefly, a Bayesian framework was used to fit thermal responses for mosquito and parasite traits that drive transmission, which were empirically estimated in laboratory experiments, and then combined to obtain the posterior distribution of *R*_*0*_ as a function of temperature. The full methods for this Bayesian approach are described in detail in Johnson et al. [37]*.* The posterior samples for *R*_*0*_ as a function of temperature (rescaled to range from zero to one, given that the absolute magnitude of *R*_*0*_ in any given setting varies) were generated, and the probability that *R*_*0*_ > 0 at each temperature was obtained, a cutoff inclusive of any transmission risk (not just sustained outbreaks, where *R*_*0*_ > 1). This is expressed as *S(T)*, the suitability for transmission as a function of temperature, and the thermal boundaries where posterior probability for *S(T)* > 0 is > 0.975 define the limits on suitability for monthly mean temperatures (as described in Ryan et al. [38]). The temperature bounds for transmission for ASPV and ASPF are 16.6–31.7 °C and 16.0–36.5 °C, respectively [29].

### Mapping malaria transmission suitability

The gridded global temperature data (baseline and future climate scenarios, month-wise) were clipped to the boundary of Nepal and constrained to the temperature limits of *S(T)* described above. Pixel-wise monthly binary suitability (1 or 0) was summed, to represent the number of months of the year for which that pixel is suitable for malaria transmission. This analysis was conducted in R (R version 4.1.3 (2022-03-10)) using “raster,” “rgdal,” “sp,” and “maptools” packages. Malaria transmission suitability (Table 1) was then categorized based on the number of months of suitability, criteria used by Ryan et al. [8]. This method generated raster files of transmission suitability for baseline, 2030 and 2050 for each RCP 4.5 and RCP 8.5 for each of ASPF and ASPV on the landscape of Nepal. Then, a series of maps were produced using ArcGIS (Version 10.6.1) to visualize the predicted changes and compared with the observation data for the DRDs, DRWs and ITCs.

**Table 1:** Definitions of malaria transmission suitability, as used by Ryan et al. [8, 39–41]

Malaria Transmission Suitability	Definition
Endemic	Malaria transmission suitable for 10–12 months of the year
Seasonal	Malaria transmission suitable for 7–9 months of the year
Moderate	Malaria transmission suitable for 4–6 months of the year
Marginal	Malaria transmission suitable for 1–3 months of the year
Unsuitable	Not suitable for malaria transmission

### Comparing malaria transmission suitability between baseline climate and predicted climate

The changes in malaria transmission suitability were compared between baseline climate and predicted climate. For example, if the Endemic suitability during baseline climate will change to other categories like Seasonal, Moderate, Marginal and Unsuitable during predicted climate.

### Comparing malaria transmission suitability between ASPF and ASPV

The malaria transmission suitability between ASPF and ASPV were compared using niche overlap similarity estimate: Warren’s I metric [42]. Warren’s I metric is often used to estimate spatial overlap of niche model predictions [43, 44]. The Warren’s I metric ranges from a value of 0 (no overlap) to 1 (perfect overlap). This metric was also used to compare malaria transmission suitability between two RCPs for both ASPF and ASPV.

### Comparing the future malaria transmission suitability with previously defined areas of risk

Using geospatial layers of DRDs, DRWs, and ITCs, these three previous definitions of risk were first compared to describe overlaps and gaps. Then, predicted malaria transmission suitability and shifts were compared with each of the DRDs, DRWs and ITCs.

## Results

### Baseline malaria suitability

**Fig. 2:**
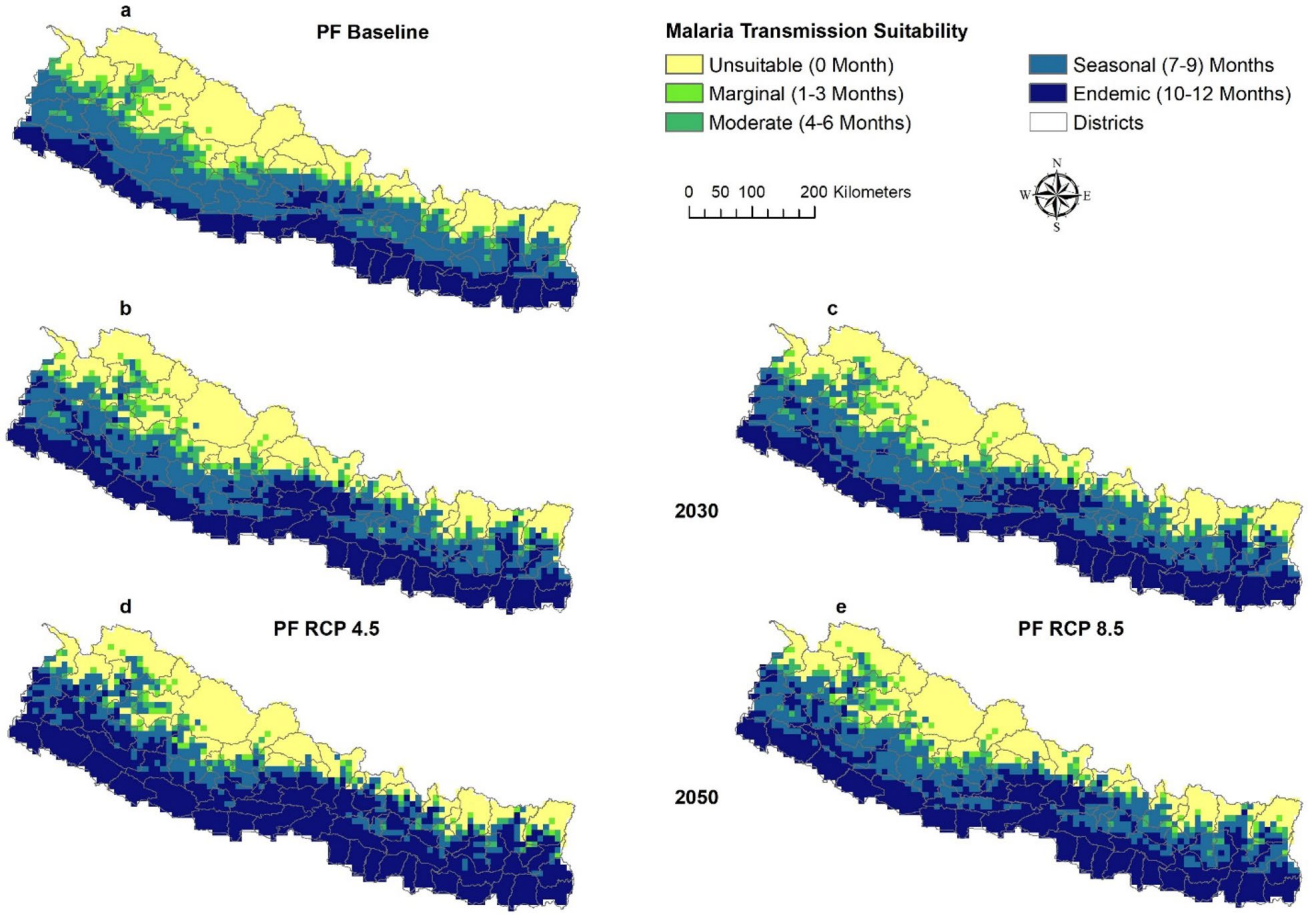
Baseline and predicted malaria transmission suitability by ASPF. The predictions were made with ensembles of four GCMs. Baseline malaria transmission suitability (**a**). Future predictions include two RCPs: 4.5 (**b** and **d** and 8.5 (**c** and **e**) for two time periods 2030 (**b**–**c**) and 2050 (**d**–**e**)

At baseline conditions (1960–1990), malaria transmission suitability for both *P. falciparum* and *P. vivax* is predicted to be endemic in the southern parts of country. The suitability decreases towards the north, with the northernmost part of the country unsuitable for malaria transmission. The baseline malaria suitability map (Fig. 2a) visually aligns with the district-level official malaria risk stratification map of 2010 (Additional file 1: Fig. S1).

### Predicted malaria suitability

#### Changes in overall malaria suitability

The results anticipate shifts in geographic and seasonality patterns for malaria transmission for ASPF and ASPV under both RCP 4.5 and RCP 8.5 (Fig. 2) in 2030 and 2050 as compared to baseline. Areas with endemic, seasonal, moderate, and marginal suitability will expand and move towards the north, towards higher altitude, while previously unsuitable areas will contract.

#### Major shifts in suitability

**Fig. 3:**
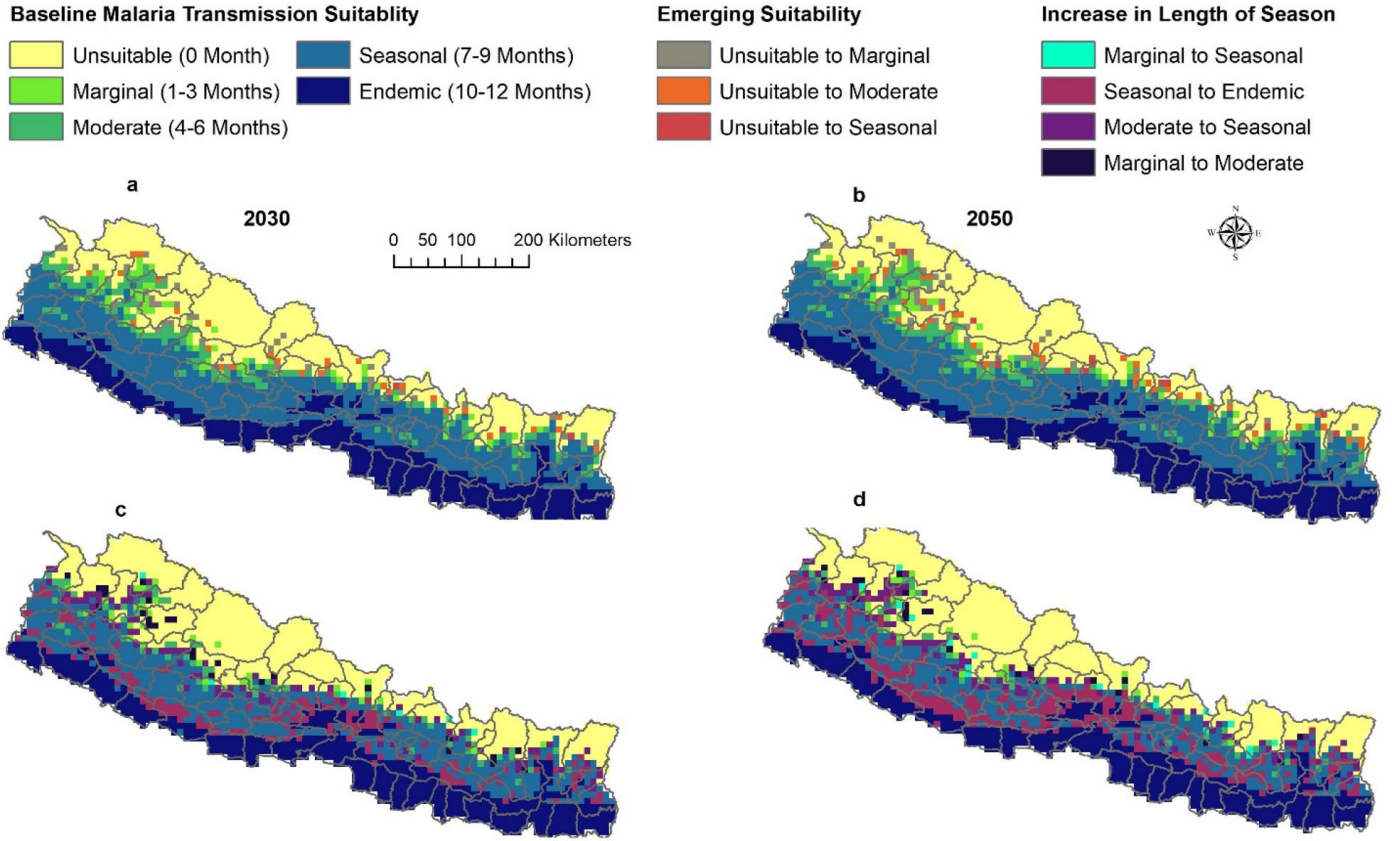
Shifting malaria transmission suitability by ASPF under RCP 8.5. The predictions were made with ensembles of four GCMs. Areas with Emerging Suitability (**a**–**b**) and Areas with Increase in Length of Season (**c**–**d**)

**Fig. 4:**
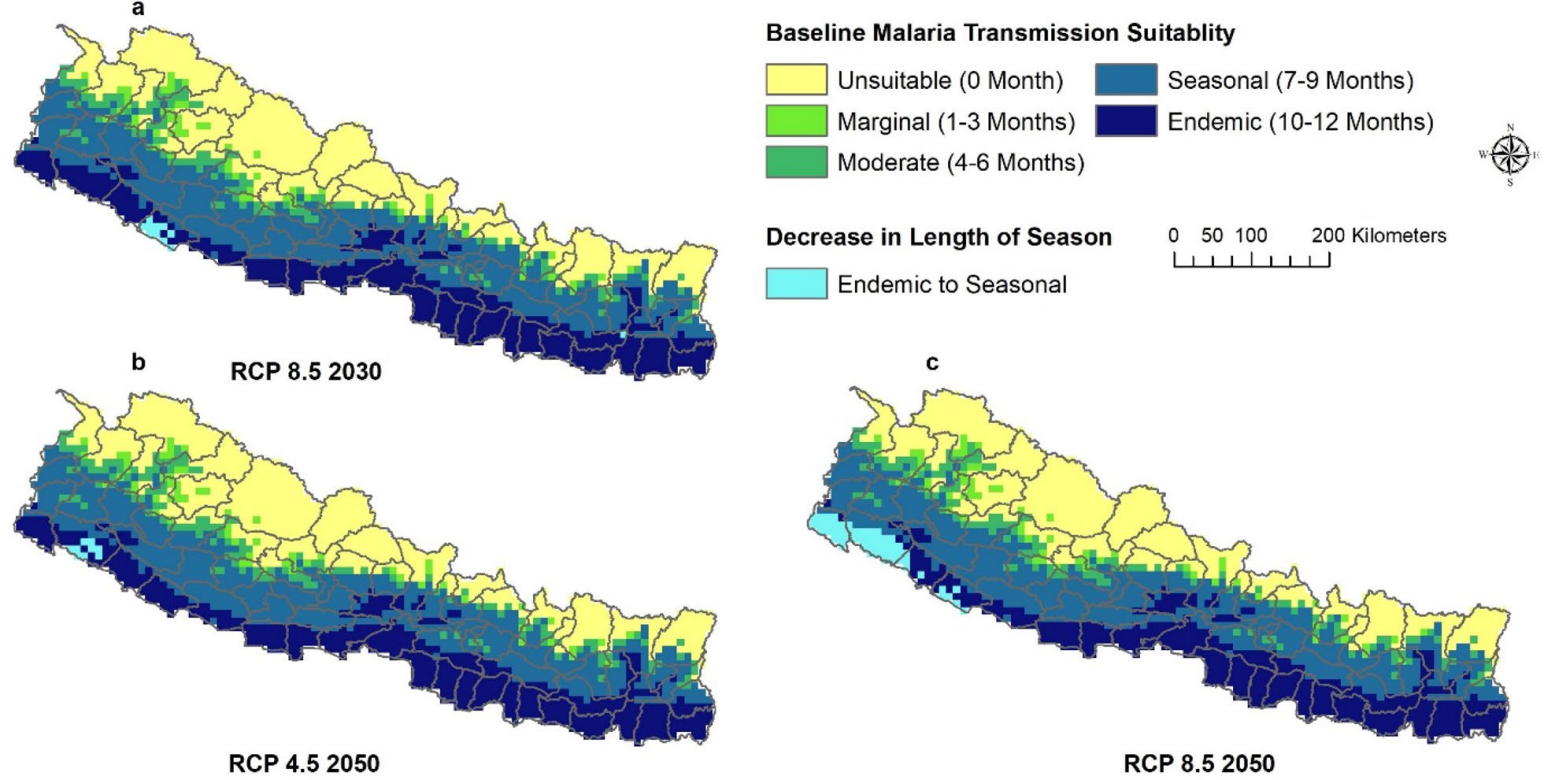
Decrease in length of season of malaria transmission by ASPV in 2030 and2050. The predictions were made with ensembles of four GCMs

Between baseline and projected future climate scenarios, bidirectional changes were anticipated between seasonal categories. For example, seasonal suitability will change to endemic suitability, moderate suitability to seasonal suitability. All potential category of shifts in suitability were evaluated and major shifts were presented as mapped outcomes (Figs. 3, 4) with additional shifts shown in a supplemental figure (Additional file 1: Fig. S2).

The shifts in suitability were described in four categories, as follows.

*Emergence of suitability* Areas unsuitable at baseline climate will become suitable for malaria transmission in the future climate scenario. Unsuitable areas are anticipated to become seasonally, moderately, and marginally suitable in 2030 and 2050 (Fig. 3a–b). Unsuitable areas will become marginally suitable mostly in the Karnali province; moderately suitable in the junction of Sudur Paschim and Karnali Province, and northern part of Bagmati and Gandaki Province; and seasonally suitable mostly in Gandaki province (Fig. 3).

*Disappearance of suitability* Some areas with moderate and marginal suitability at baseline, will become unsuitable for malaria transmission in the future. Those shifts occurred in very few places scattered across the country (Additional file 1: Figs. S2, S3).

*Increase in length of season* The length of season for malaria transmission suitability will increase in some areas. Marginal suitability will become moderately, and seasonally suitable, moderately suitable areas will become seasonally suitable and seasonally suitable areas will become endemically suitable (Fig. 3c–d).

The most predominant shift between baseline and predicted future climate scenarios is that vast areas predicted to have seasonal suitability at baseline will become areas with endemic suitability. As a result, endemic suitability will expand into the north of Nepal. The expansion of endemic suitability will be scattered throughout the country from east to west in all provinces. This expansion will become more prominent in the 2050. Similarly, seasonal suitability will also shift towards the north. Most of this shift will occur in the junction of Sudur Pashchim and Karnali province, and junction of Lumbini and Gandaki province. Marginal suitability will also shift to moderate suitability, and this will occur mostly in the junction of Sudur Pashchim and Karnali province. Marginal suitability will shift into seasonal suitability at the junction of Sudur Paschim and Karnali Province, and the junction of Karnali and Lumbini Province.

*Decrease in length of season *Some areas are predicted to experience a decrease in the length of season for malaria transmission in the future. The endemic suitability will change into seasonal suitability, seasonal into moderate, and moderate into marginal suitability. However, these changes are anticipated to occur in very few places scattered across the country (Additional file 1: Figs. S2, S3) except for ASPV where areas with endemic suitability will change into seasonal suitability (during 2030 under RCP 4.5 and RCP 8.5 but only during 2050 under RCP 4.5) in the southwestern part of the country concentrated in few areas in Sudur Paschim and Lumbini Province (Fig. 4).

### Comparing malaria transmission suitability between ASPF and ASPV

There will be some differences in malaria transmission suitability between baseline and future predictions for ASPF and ASPV besides the decreasing in length of season in Fig. 4. Some change in categories will not happen for either of them in different years under different RCPs. For example, for ASPF, moderate areas will convert into unsuitable areas in all scenarios. For ASPV, moderate areas will convert into unsuitable areas only during 2030s, but not during 2050s. All the differences in change in categories are placed in Additional file 1: Table S2.

The similarity of malaria transmission suitability between ASPF and ASPV, and between RCPs and scenario years within ASPF and ASPV predictions, were also compared using Warren’s I metric. Warren’s I metric values for all comparisons were greater than 0.9, indicating a high degree of overlap (Additional file 1: Table S1). This means that there is not much difference in predicted malaria transmission suitability distribution between ASPF and ASPV, across RCPs 4.5 and 8.5, and between 2030 and 2050. Additionally, this implies that the changes in geographic and seasonality of malaria transmission suitability is broadly consistent across both scenarios of future climate for both ASPF and ASPV, thus the maps of only ASPF are presented in the main results. Maps of ASPV are in Supplemental findings (Additional file 1: Figs. S4, S5). Similarly, results for RCP 8.5 are presented in the main results and for RCP 4.5 in the Supplemental findings (Additional file 1: Figs. S6, S7).

### Comparison of previously defined spatial malaria risk descriptors

#### Incidence trend clusters (ITCs), 2010 DRDs and 2021 DRWs

**Fig. 5:**
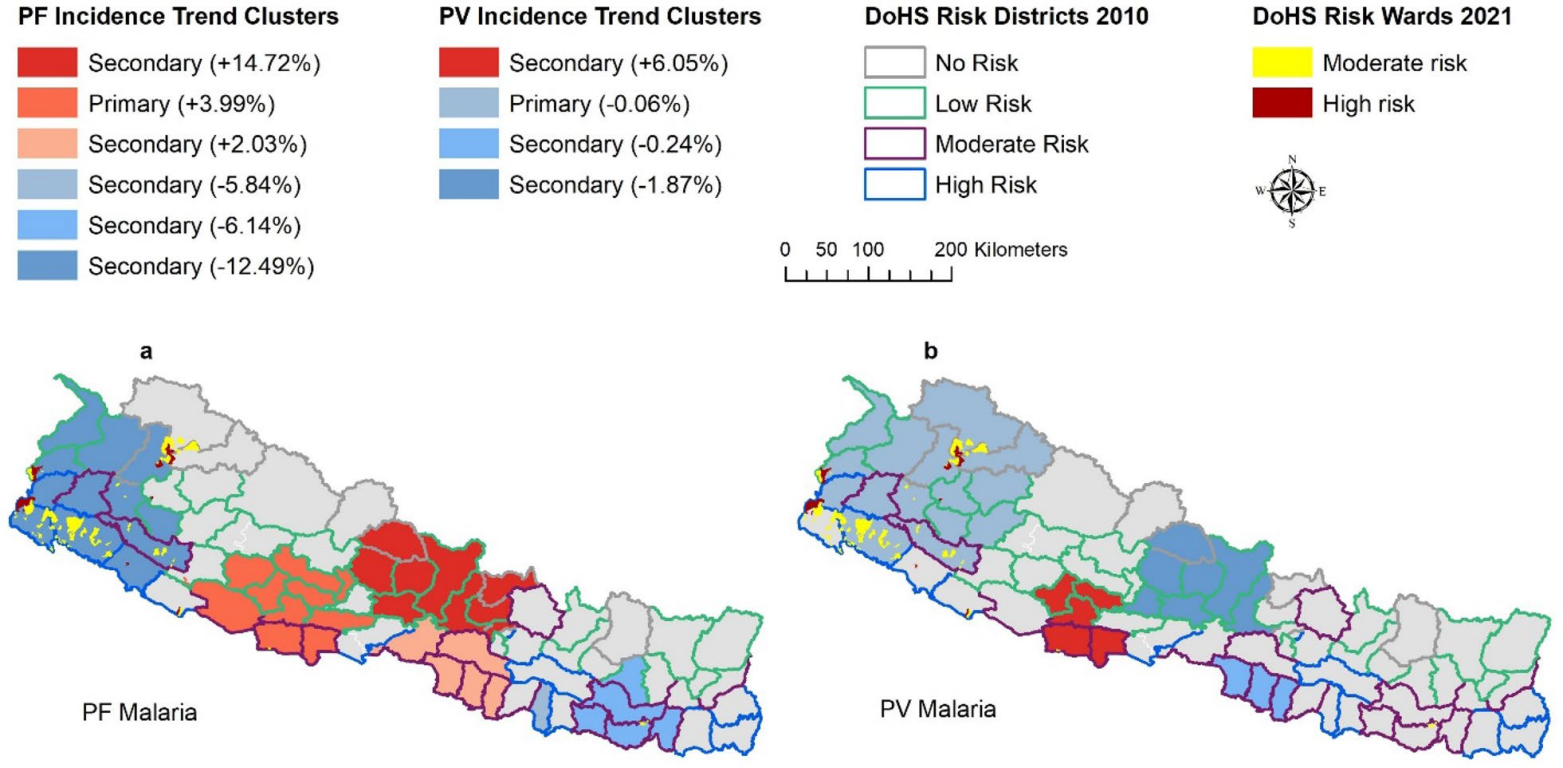
Incidence trend clusters comparison with DoHS risk wards. PF ITCs (**a**) and PV ITCs (**b**). Red clusters (positive values) reflect increasing ITC rates within districts and blue clusters reflect decreasing rates (negative values)

The ITCs with increasing trends of PF malaria include moderate risk, low-risk, and no-risk 2010 DRDs (Fig. 5a). Similarly, the ITCs with increasing trends of PV malaria include moderate and low-risk 2010 DRDs (Fig. 5b). Moreover, the high and moderate-risk 2021 DRWs fall into ITCs with decreasing trend but not in the ITCs with increasing trend of PF and PV malaria (Fig. 5).

#### Predicted future suitability, 2010 DRDs, and 2021 DRWs

**Fig. 6:**
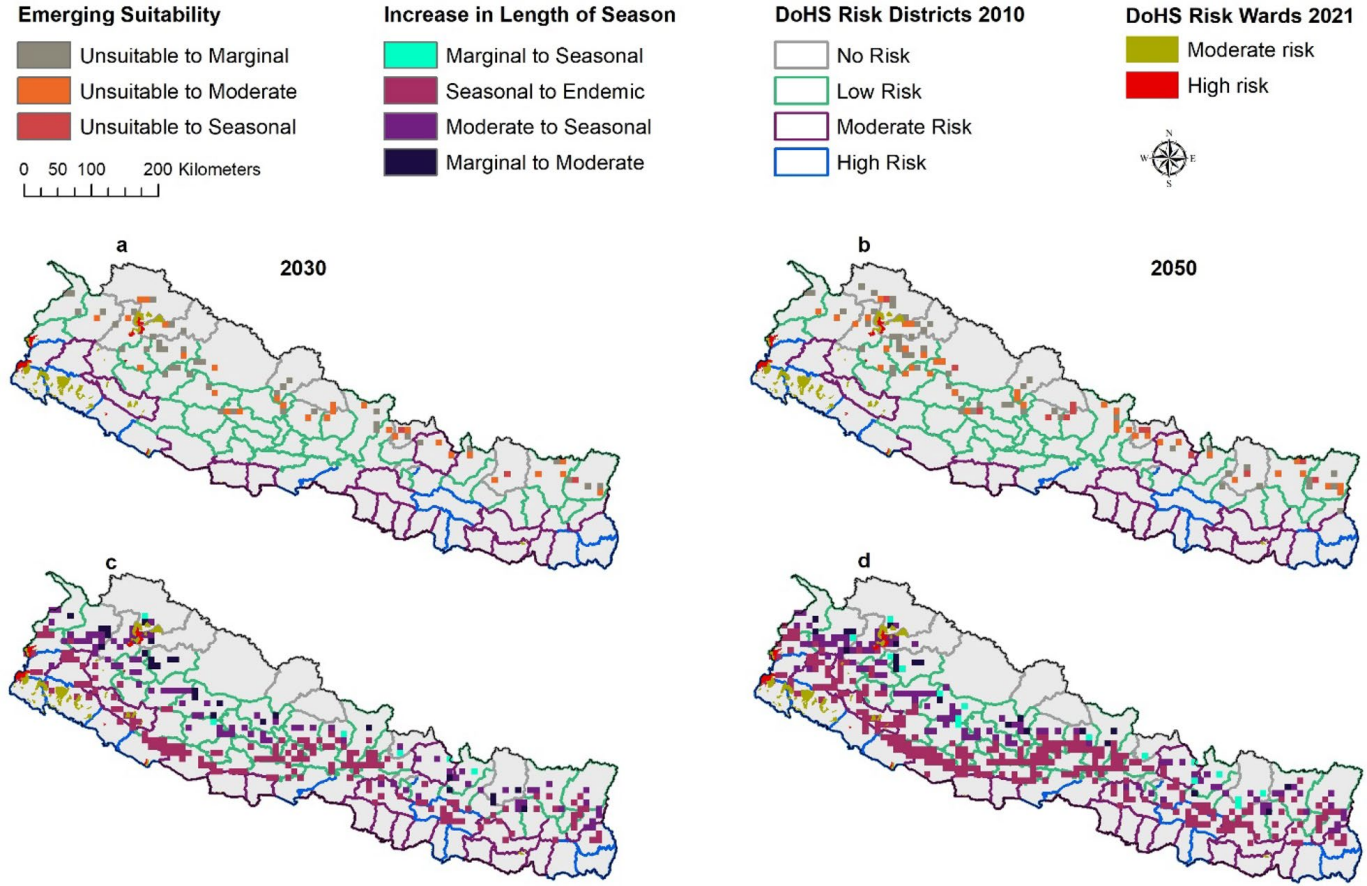
Shifting malaria transmission suitability by ASPF under RCP 8.5 and 2010 DRDs and 2021 DRWs. The predictions were made with ensembles of four GCMs. Areas with Emerging Suitability (**a**–**b**) and Areas with Increase in Length of Season (**c**–**d**)

Emergence of suitability is predicted to occur in the low and no-risk 2010 DRDs as unsuitable areas become marginally, moderately, and seasonally suitable Fig. 6a–b). Seasonal suitability will expand into endemic suitability in the high, moderate, and low- risk 2010 DRDs (Fig. 6c–d). Moderate suitability will expand into seasonal suitability mostly in the low-risk and no-risk DRDs; similarly, marginal suitability will expand into moderate suitability and seasonal suitability in low-risk and no-risk DRDs. Thus, an increase in length of season will occur across all 2010 DRD categories.

When comparing the future predicted suitability with the 2021 DRWs, it was found that high and moderate-risk 2021 DRWs do not fall into unsuitable areas converting into suitable areas (Fig. 6a–b). Thus, anticipated emergence of transmission suitability is outside the designated high and moderate-risk DRWs. Similarly, very few areas with increase in length of season such as seasonal to endemic, and moderate to seasonal encompass the high and moderate-risk 2021 DRWs (Fig. 6c–d). Most of the increase in length of season will occur outside of the high and moderate-risk 2021 DRWs.

#### Predicted future suitability and ITCs

**Fig. 7:**
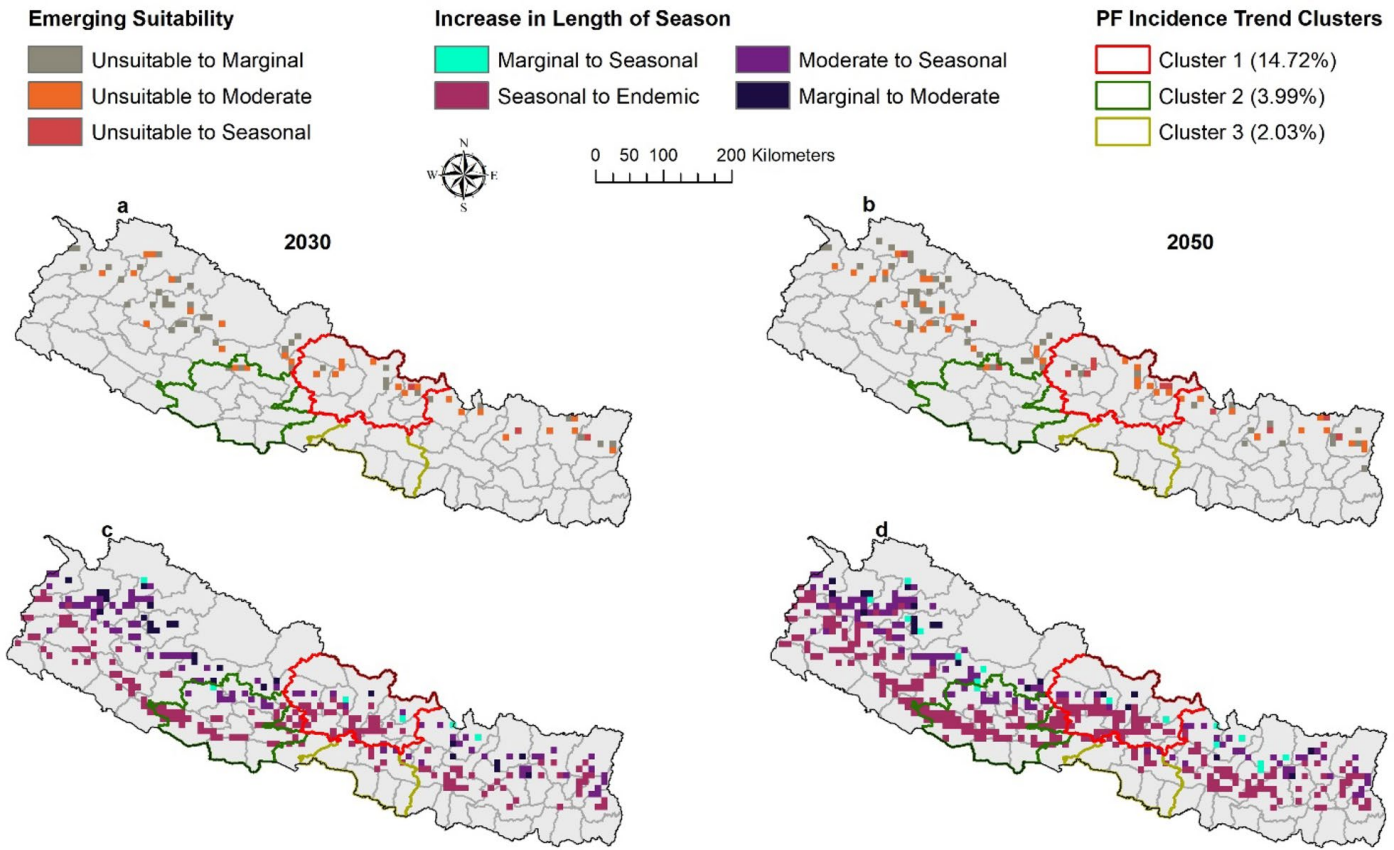
Shifting malaria transmission suitability by ASPF under RCP 8.5 and comparing with ITCs of PF malaria. The predictions were made with ensembles of four GCMs. Areas with Emerging Suitability (**a**–**b**) and Areas with Increase in Length of Season (**c**–**d**)

**Fig. 8:**
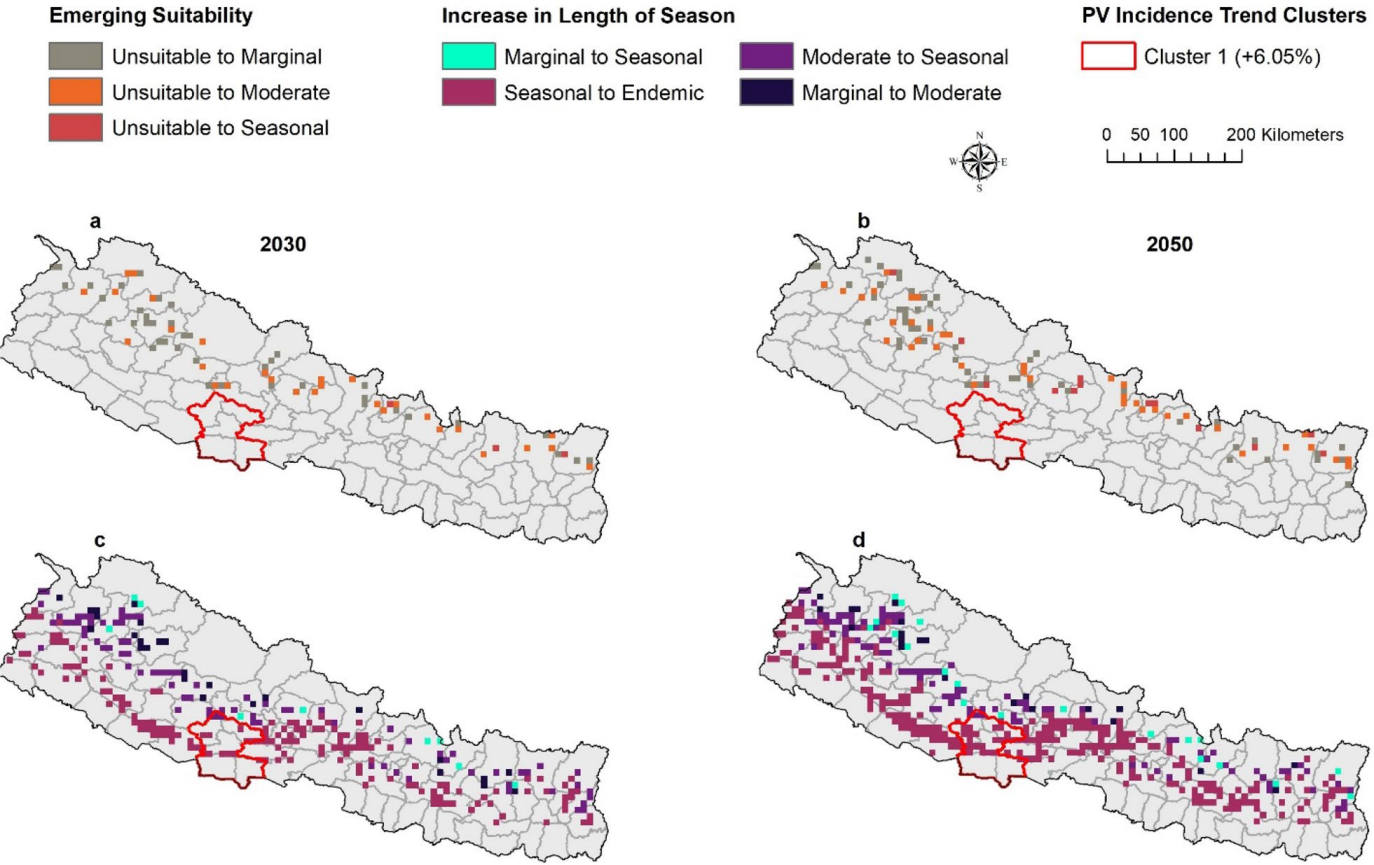
Shifting malaria transmission suitability by ASPV under RCP 8.5 and comparing with ITCs of PV malaria. The predictions were made with ensembles of four GCMs. Areas with Emerging Suitability (**a**–**b**) and Areas with Increase in Length of Season (**c**–**d**)

Areas with predicted emerging suitability fall inside PF ITCs (Figs. 7a–b). Unsuitable areas converting to marginal and moderate suitability will fall inside Clusters 1 and 2. Unsuitable areas converting to seasonal suitability will fall inside Cluster 1. Similarly, the areas with the increasing length of season fall inside ITCs of increasing PF trend (Fig. 7c–d). Areas with seasonal suitability converting into endemic suitability, and moderate suitability converting into seasonal suitability fall inside Clusters 1, 2 and 3. Areas with marginal suitability converting to moderate suitability fall inside ITC cluster 1 and marginal suitability converting into seasonal suitability in clusters 1 and 2. Emergence of suitability will occur in Clusters 1 and 2 while Increasing length of season will occur in all three clusters. Similarly, only areas of seasonal suitability converting into endemic suitability fall inside an increasing PV ITC (Fig. 8c–d).

## Discussion

This study assessed baseline and future geographic distribution of malaria transmission suitability in Nepal and compared the anticipated future distribution with previously defined areas of malaria risk from two different approaches.

This study found the predicted geographic distribution of malaria transmission suitability of both ASPF and ASPV will increase in the future. For ASPV, in addition to increase, there will be decrease in suitability in all scenarios except in 2030 under RCP 4.5 A study by Hundessa et al. found that the percentage of *P. vivax* and *P. falciparum* malaria will increase in China in 2030s, 2050s and 2080s under RCP 4.5 and 8.5 scenario [45]. In addition, the study found that the percent increase of *P. falciparum* malaria will be higher than for *P. vivax* malaria and their spatial distributions will differ. In this study, the predicted geographic distribution is nearly the same for both ASPV and ASPF according to the Warren’s I metric.

In this study, the geographical distribution of malaria transmission suitability in 2030 and 2050 did vary compared to baseline. As compared to baseline, there will be emergence of suitability and increase in length of Season for malaria transmission for both ASPF and ASPV in 2030 and 2050, except for ASPV, where in addition to emergence of suitability and increasing length of season, some areas will observe decreasing length of season (endemic to seasonal) in the southwestern part of the country. A similar study carried out for Africa also found that climate change will result in shifting of geographic distribution of malaria transmission suitability in the years 2030, 2050, and 2080 under RCP 4.5 and 8.5 [8]. According to the study, large areas previously unsuitable for malaria transmission in Africa will convert into areas with endemic and seasonal suitability as well as areas that shift from endemic or seasonal suitability to becoming unsuitable for malaria transmission.

Malaria transmission suitability is predicted to emerge and increase in length of season in previously cooler regions of Nepal, towards the northern part of the country in the higher altitudes. This means that malaria will be able to establish in these areas, and local transmission, not just imported cases, can occur. These regions were less suitable or not suitable for malaria transmission in the past. Due to climate change, temperatures will rise, and these regions will become more suitable for malaria transmission. Several other studies have found that malaria already spread in previously cooler places and extended towards higher altitude in various parts of the world. For example, increased number of malaria cases were found at higher altitudes in the highlands of Colombia during warmer years [19]. In Rwanda, there was a substantial increase in malaria cases country-wide, but the rate of increase was greater at high elevations than at medium and low elevations [18]. In Rwanda, malaria migrated to new areas in the late 1980s, to places where it was previously rare or absent due to record high temperatures and heavy rains in 1987 followed by the El Nino event in 1988 [18]. Similarly, some studies have predicted increase in malaria in previously cooler places. For example, Hundessa et al. predicted that both *P. vivax* and *P. falciparum* malaria will increase in the previously cooler regions in China in the future under climate change [45]. Similarly, Ryan et al. predicted that due to climate change, exposure to malaria transmission will increase in previously unsuitable regions such as the higher elevation regions of Southern and Eastern Africa in 2030 and will become more concentrated along the Eastern African highlands later (2080) [8].

The results of this study have identified regions with the emergence of suitability, increasing length of season and decreasing length of season, where interventions need to be revisited given the impacts of future climate change. With increase in temperature due to climate change, malaria transmission is expected to occur in some previously unsuitable regions (emergence of suitability), for example in the Karnali province, junction of Sudur Paschim and Karnali province, northern part of Bagmati and Gandaki province, particularly in the Mountainous region of Nepal. The emergence of suitability will put naive population at risk of outbreaks, especially the vulnerable groups such as pregnant women, children, and the elderly. These places need a new malaria control program. Similarly, malaria transmission suitability is expected to increase (increasing length of season) in other previously suitable regions, for example in the junction of Sudur Paschim and Karnali province, Lumbini, Gandaki and Bagmati province, in the Hilly and Mountainous region in Nepal. In these places, malaria seasons are getting longer. This will require different control interventions and management activities than those currently in practice for a shorter malaria season. On the other hand, malaria transmission suitability for ASPV will decrease from endemic to seasonal (decreasing length of season) in the Terai region in Nepal. In these areas, opportunities arise for more targeted intervention and elimination of the disease.

In this study, the changing areas of malaria risk in future were compared with the previously defined areas of risk. First, DoHS risk areas of 2010 and 2021 were compared with the incidence trend clusters from our previous study. ITCs with increasing PF and PV malaria encompass moderate-risk, low-risk, and no-risk 2010 DRDs, but do not encompass high-risk districts. This means that between 2005 and 2018, the PF and PV malaria had increasing trend in some of the moderate, low and no-risk districts and not in the high-risk districts and it may be because vector control interventions were mostly focused on high-risk districts followed by moderate risk districts with little or no interventions in the low and no-risk districts. Comparing the ITCs with DoHS risk wards of 2021, it was found that the high and moderate-risk wards of 2021 do not lie in the ITCs with increasing trend of PF and PV malaria, but they lie in the ITCs with decreasing trend of PF and PV malaria. The increasing trend of PF and PV malaria are forming in places where vector controls are not implemented. Public health officials can use the information of increasing trend of PF and PV malaria and make plans for implementing vector control in those clusters.

It was also found that the emergence of suitability will occur in low and no-risk DRDs and outside of the high and moderate-risk DRWs. Increasing length of season will occur in all categories of DRDs and only few areas with increasing length of season will occur in high and moderate-risk DRWs but mostly outside of them. Thus, this study has identified potential malaria risk areas in the future which are outside of the government’s designated malaria risk areas and thus not in attention of the public health officials for vector control interventions. As, the malaria risk stratification by the government has not included the impact of climate change in their risk designations [27], the results of this study can be helpful in planning for additional areas for vector control interventions.

Similarly, emergence of suitability and increasing length of season are predicted in PF and PV ITCs. This may indicate that the increasing trend of PF and PV malaria is an impact of climate change already affecting malaria transmission in Nepal, which will increase further in the future. Thus, not only are areas of malaria risk already changing in Nepal but will continue to in the future with climate change. Thus, public health officials may need to revisit official risk stratification mapping for the future, incorporating the potential impacts of climate change. Nepal is currently preparing for malaria elimination by 2026, and this may be feasible. However, there is also always the potential for malaria resurgence following elimination because the risk factors for malaria like suitable climate and migration of people (who can introduce imported malaria) still exists. Thus, these potential future scenarios of malaria transmission suitability (emergence, disappearance, increase and decrease) should be incorporated into the planning of vector control interventions and other malaria management programmes, to elimination and beyond. Otherwise, the gains achieved in malaria control in recent decades could be lost.

This study availed itself of the best data accessible to the authors, but parts of the information presented may have been limited by data availability in several aspects of the project. The authors note that after adopting new constitution in 2015, Nepal underwent restructuring of its administrative divisions into 7 provinces, 77 districts, and 753 municipalities and rural municipalities [46, 47]. Before 2015, Nepal was administratively divided into 5 development regions, 14 zones, 75 districts, 53 municipalities, and 3,918 village development committees (VDCs) [48, 49]. In this study, the 75-district presentation was retained. For this study, we are using ward level data for comparison based on the administrative division after 2015. However, for the incidence trend clusters (ITCs), malaria data for 75 districts were used, based on administrative divisions prior to 2015 because epidemiological data was available for 75 districts for most years between 2005 and 2018. The before and after 2015 maps of districts are presented in Additional file 1: Fig. S8 the subdivision of two previous districts did not affect the findings of this study.

The malaria burden data used by Nepal DoHS only include malaria surveillance data from the public health facilities, while malaria information from private sector healthcare is unreported [50]. Thus, the malaria burden data may not represent the actual malaria transmission situation on the ground, which may have affected the official microstratification definitions. The government should focus on strategizing to access a greater proportion of malaria testing information, including prioritizing policy making for including private health facilities in the malaria reporting system. The potential limits to the full geographic scope of Nepal’s reported malaria burden meant that groundtruth models could not be performed on the reported risk definitions. The places where alignment or mismatches occurred could only be commented upon.

The results of this study are based on the temperature response curves of *An. stephensi* and the malaria parasites because there are not enough studies that have conducted laboratory experiments for identifying thermal responses of *An. fluviatilis*, the major malaria vector in Nepal. The studies on thermal responses of mosquitoes are mostly for species that are found in Africa for example *Anopheles gambiae* and *An. stephensi* [29]. There is a need of more studies on thermal responses of *Anopheles* species found outside of Africa (for example *fluviatilis*) because malaria is a major issue in Asia after Africa and *An. fluviatilis* is a major vector in countries like India, Nepal, Iran, Pakistan, Afghanistan, Bangladesh, and Myanmar [12]. As the entire world is gearing up for elimination of malaria, more information is needed on how climate change might impact the malaria transmission for major malaria vectors not only in Africa but also outside of Africa. In addition, more mosquito surveillance is needed in Nepal to timely update the malaria vectors.

*Anopheles stephensi* has been reported to be present in Nepal in survey studies [51, 52], and is assumed to be part of the suite of competent malaria vectors. However, a recent entomological study conducted in the eastern part of Nepal did not report *An. stephensi* [32], and we are not aware of any more recent published or publicly available mosquito survey studies in Nepal in recent years. The absence of records at present are due to under-surveyed conditions, rather than a reflection of its absence, and suggest that understanding the potential for *An. stephensi* to expand malaria risk in Nepal is an important part of the malaria management strategy for the country.

The results of this study are based on the impact of temperature on malaria. Precipitation, another climatic factor which influences malaria transmission was not included, in this study. Mosquitoes require water as breeding habitats to complete their life cycle. However, precipitation measures such as monthly rainfall totals or cumulative rainfall may not be a good indicator of standing water, as extreme precipitation events are becoming more common with climate change [8]. Heavy rainfall can wash away the mosquito breeding sites disrupting their life cycle. Thus, more rain may not mean more breeding sites and more mosquitoes. In addition, there are more uncertainties in predicting future precipitation with climate change [53].

## Conclusion

This study presented the predicted impact of climate change on geographical distribution and seasonality of malaria transmission suitability by *An. stephensi* in the future in Nepal and compared mapped results with mapped reported officially designated areas of malaria risk, used by Nepal’s department of health services in planning intervention and vector control activities. The study identified areas where malaria transmission suitability will emerge (Karnali province, junction of Sudur Pashchim and Karnali Province, northern part of Bagmati and Gandaki province), disappear (few areas scattered across the country), increase (junction of Sudur Paschim and Karnali province, Lumbini, Gandaki and Bagmati province) and decrease (south-western part of Terai) in the future. It was shown that there are new areas in Nepal where malaria is already increasing and predicted to increase in the future which are outside of the public health officials’ radar for vector control intervention. This may have significant implications for Nepal’s malaria elimination efforts.

This study has improved the understanding of potential future scenarios of malaria transmission suitability in Nepal. The information about the future geographic distribution and seasonality of malaria transmission suitability can help public health decision makers plan for better malaria management programs. The surveillance system should be enhanced, including in the area currently malaria free as they are projected to have malaria in the future.

## Supplementary Information

**Additional file 1: Fig. S1.** DoHS Risk Districts 2010. **Fig S2.** Shifting malaria transmission suitability by ASPF under RCP 8.5. Areas with Disappearing Suitability **a**–**b** and Areas with Decrease in Length of Season **c**–**d**. **Fig S3.** Shifting malaria transmission suitability by ASPV under RCP 8.5. Areas with Disappearing Suitability **a**–**b** and Areas with Decrease in Length of Season **c**–**d**. **Fig S4.** Baseline and predicted malaria transmission suitability by ASPV. Baseline malaria transmission suitability **a**. Future predictions include two RCPs: 4.5 (**b** and **d** and 8.5 (**c** and **e**) for two time periods 2030 **b**–**c** and 2050 **d**–**e**. **Fig S5.** Shifting malaria transmission suitability by ASPV under RCP 8.5. Areas with Emerging Suitability **a**–**b** and Areas with Increase in Length of Season **c**–**d**. **Fig S6.** Shifting malaria transmission suitability by ASPF under RCP 4.5. Areas with Emerging Suitability **a**–**b** and Areas with Increase in Length of Season **c**–**d**. **Fig S7.** Shifting malaria transmission suitability by ASPV under RCP 4.5. Areas with Emerging Suitability **a**–**b** and Areas with Increase in Length of Season **c**–**d**. **Fig S8.** Newly formed districts in Nepal after 2015. **Table S1.** Warren’s I similarity metrics for comparing malaria transmission suitability between ASPF and ASPV. **Table S2.** Differences between malaria transmission suitability by ASPF and ASPV.

## Data Availability

The data used in this study are publicly available data. Baseline climate data were downloaded from WorldClim version 1.4 https://worldclim.org/ and future climate data were downloaded from research program on Climate Change, Agriculture, and Food Security (CCAFS) web portal (http://ccafs-climate.org/data_spatial_downscaling/). DRDs and DRWs were obtained from the reports of Department of Health Services (DoHs), downloaded from the website of Epidemiology and Disease Control Division (EDCD) of Department of Health Services, Government of Nepal (http://www.edcd.gov.np/). Data for ITCs: Malaria data were obtained from the annual reports of Department of Health Services (DoHs), downloaded from the website of Epidemiology and Disease Control Division (EDCD) of Department of Health Services, Government of Nepal (http://www.edcd.gov.np/). Census data were obtained from Census report 2014 downloaded from the website of Central Bureau of Statistics (CBS) (https://censusnepal.cbs.gov.np/Home/Index/EN). The data can also be available from the author upon reasonable request.
