# Supplementary material for: Malaria transmission in Nepal under climate change: anticipated shifts in extent and season, and comparison with risk definitions for intervention
Source: Malar J. 2022 Dec 22;21:390. doi: 10.1186/s12936-022-04417-x (PMC9773623; doi:10.1186/s12936-022-04417-x)
Supplement: Supplementary file 1 — Additional file 1: Fig. S1. DoHS Risk Districts 2010. Fig S2. Shifting malaria transmission suitability by ASPF under RCP 8.5. Areas with Disappearing Suitability a–b and Areas with Decrease in Length of Season c–d. Fig S3. Shifting malaria transmission suitability by ASPV under RCP 8.5. Areas with Disappearing Suitability a–b and Areas with Decrease in Length of Season c–d. Fig S4. Baseline and predicted malaria transmission suitability by ASPV. Baseline malaria transmission suitability a. Future predictions include two RCPs: 4.5 (b and d and 8.5 (c and e) for two time periods 2030 b–c and 2050 d–e. Fig S5. Shifting malaria transmission suitability by ASPV under RCP 8.5. Areas with Emerging Suitability a–b and Areas with Increase in Length of Season c–d. Fig S6. Shifting malaria transmission suitability by ASPF under RCP 4.5. Areas with Emerging Suitability a–b and Areas with Increase in Length of Season c–d. Fig S7. Shifting malaria transmission suitability by ASPV under RCP 4.5. Areas with Emerging Suitability a–b and Areas with Increase in Length of Season c–d. Fig S8. Newly formed districts in Nepal after 2015. Table S1. Warren’s I similarity metrics for comparing malaria transmission suitability between ASPF and ASPV. Table S2. Differences between malaria transmission suitability by ASPF and ASPV. [file 12936_2022_4417_MOESM1_ESM.docx]

## Supplemental Figures


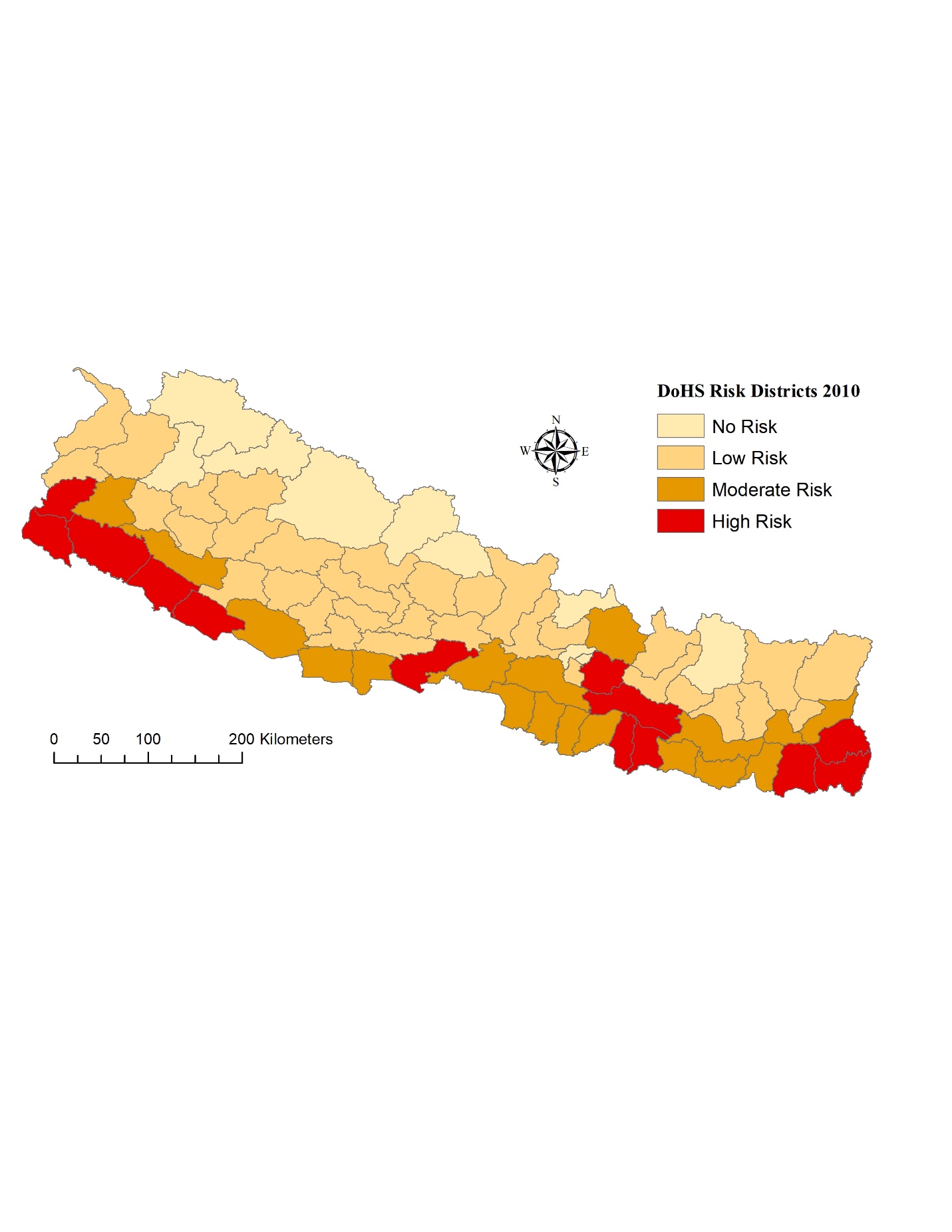


**Fig. S1: DoHS Risk Districts 2010.**


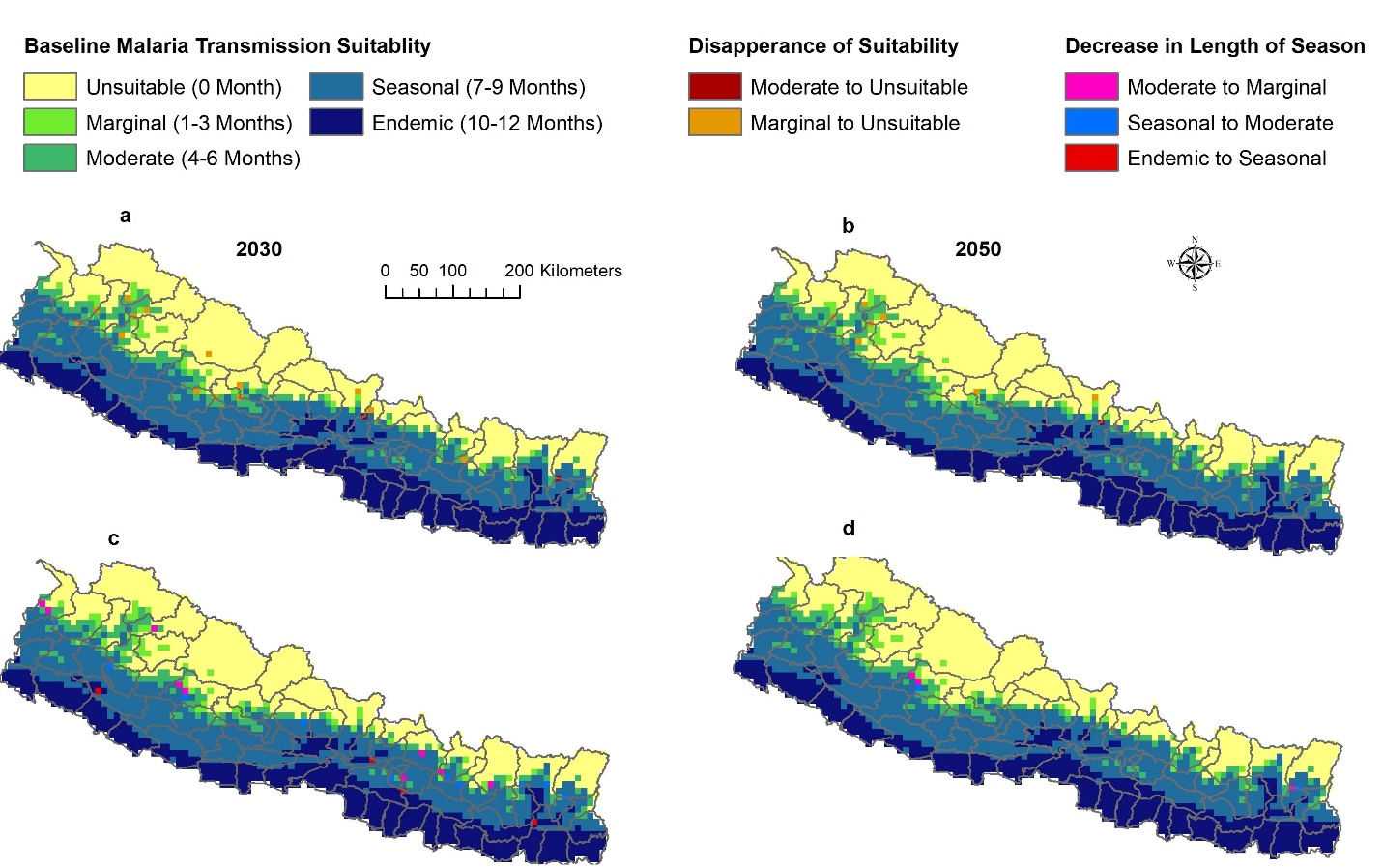


**Fig. S2: Shifting malaria transmission suitability by ASPF under RCP 8.5.** Areas with Disappearing Suitability (a-b) and Areas with Decrease in Length of Season (c-d).


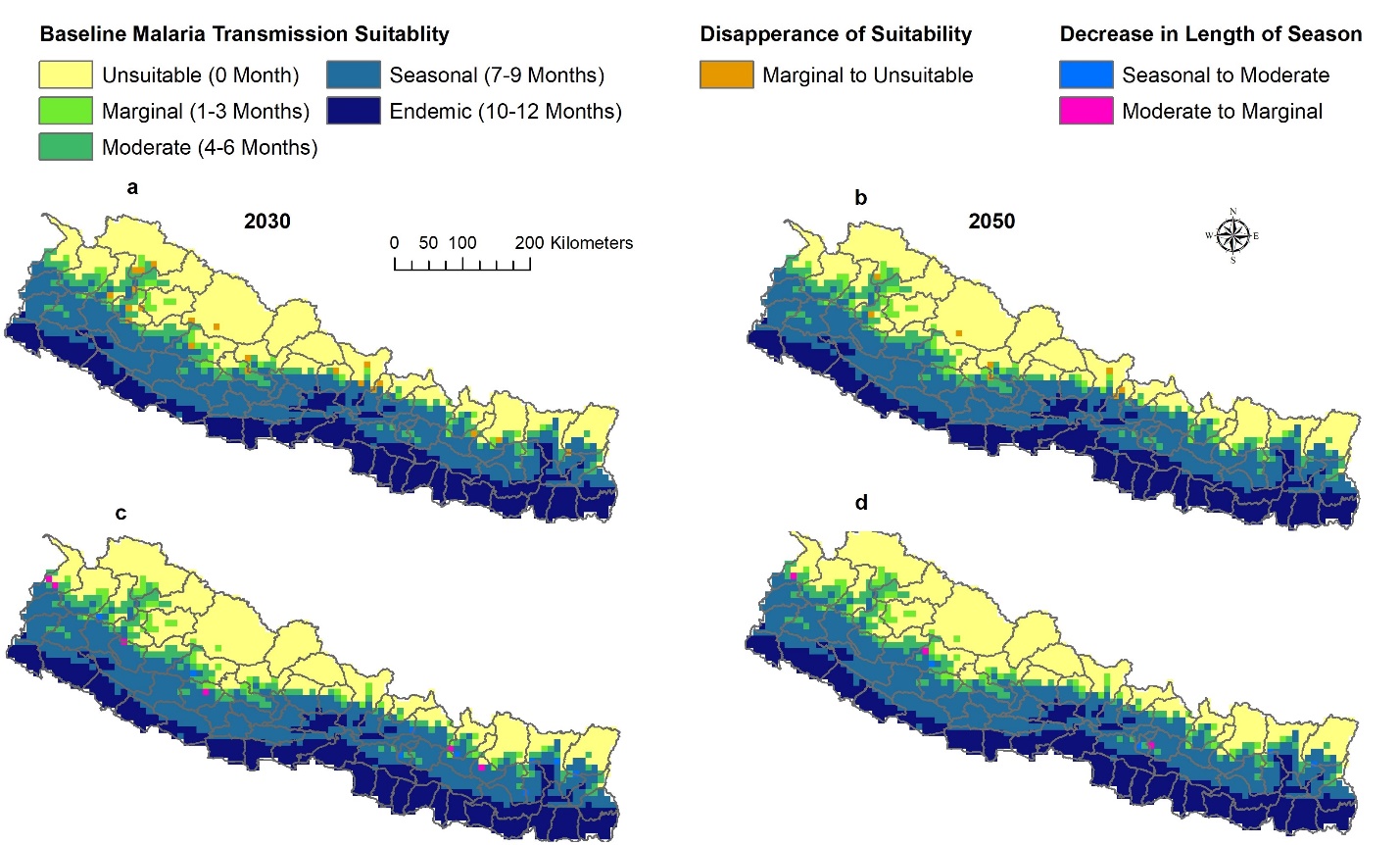


**Fig. S3: Shifting malaria transmission suitability by ASPV under RCP 8.5.** Areas with Disappearing Suitability (a-b) and Areas with Decrease in Length of Season (c-d).


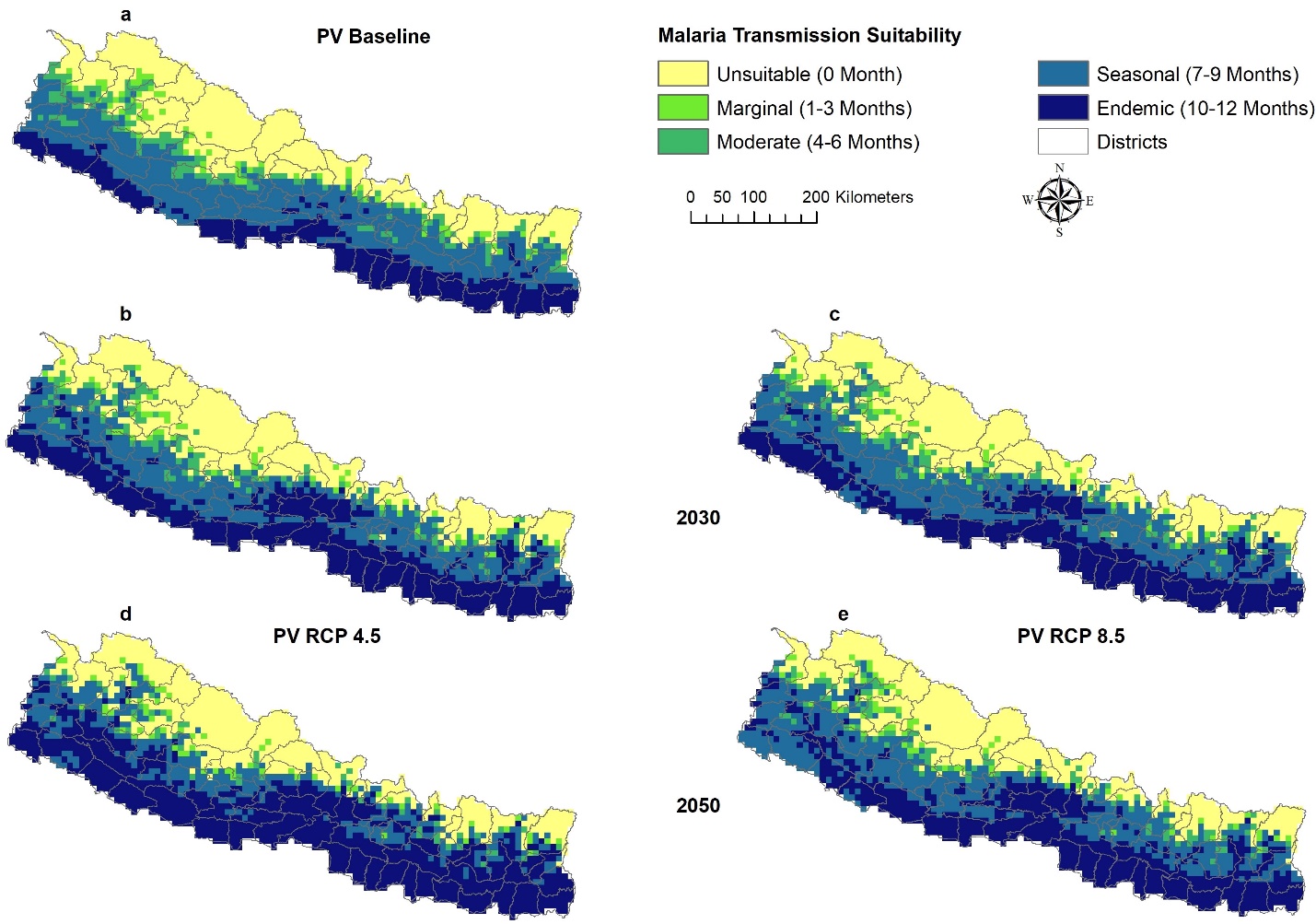


**Fig. S4: Baseline and predicted malaria transmission suitability by ASPV.** Baseline malaria transmission suitability (a). Future predictions include two RCPs: 4.5 (b and d and 8.5 (c and e) for two time periods 2030 (b-c) and 2050 (d-e).


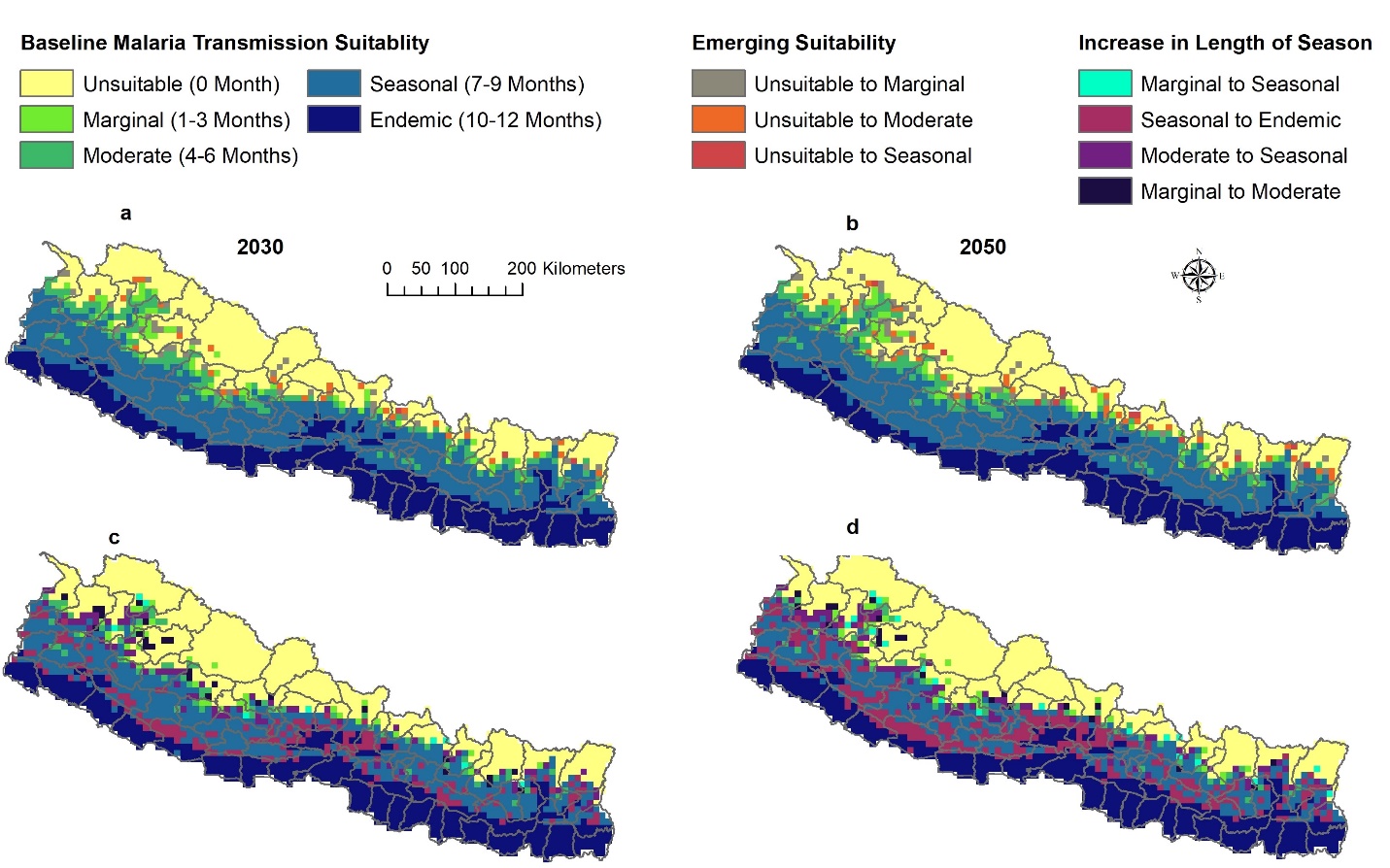


**Fig. S5: Shifting malaria transmission suitability by ASPV under RCP 8.5.** Areas with Emerging Suitability (a-b) and Areas with Increase in Length of Season (c-d).


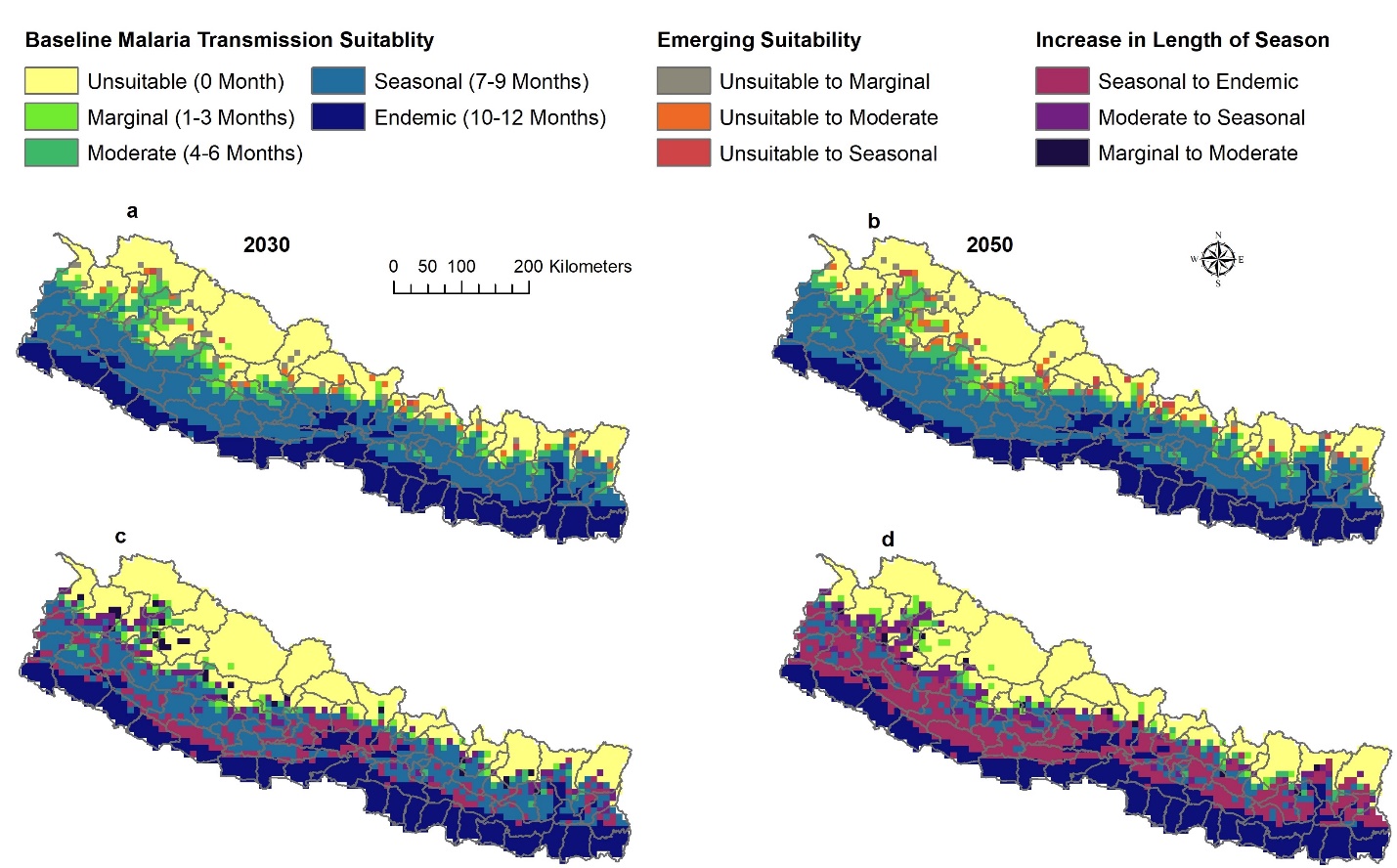


**Fig. S6: Shifting malaria transmission suitability by ASPF under RCP 4.5.** Areas with Emerging Suitability (a-b) and Areas with Increase in Length of Season (c-d).


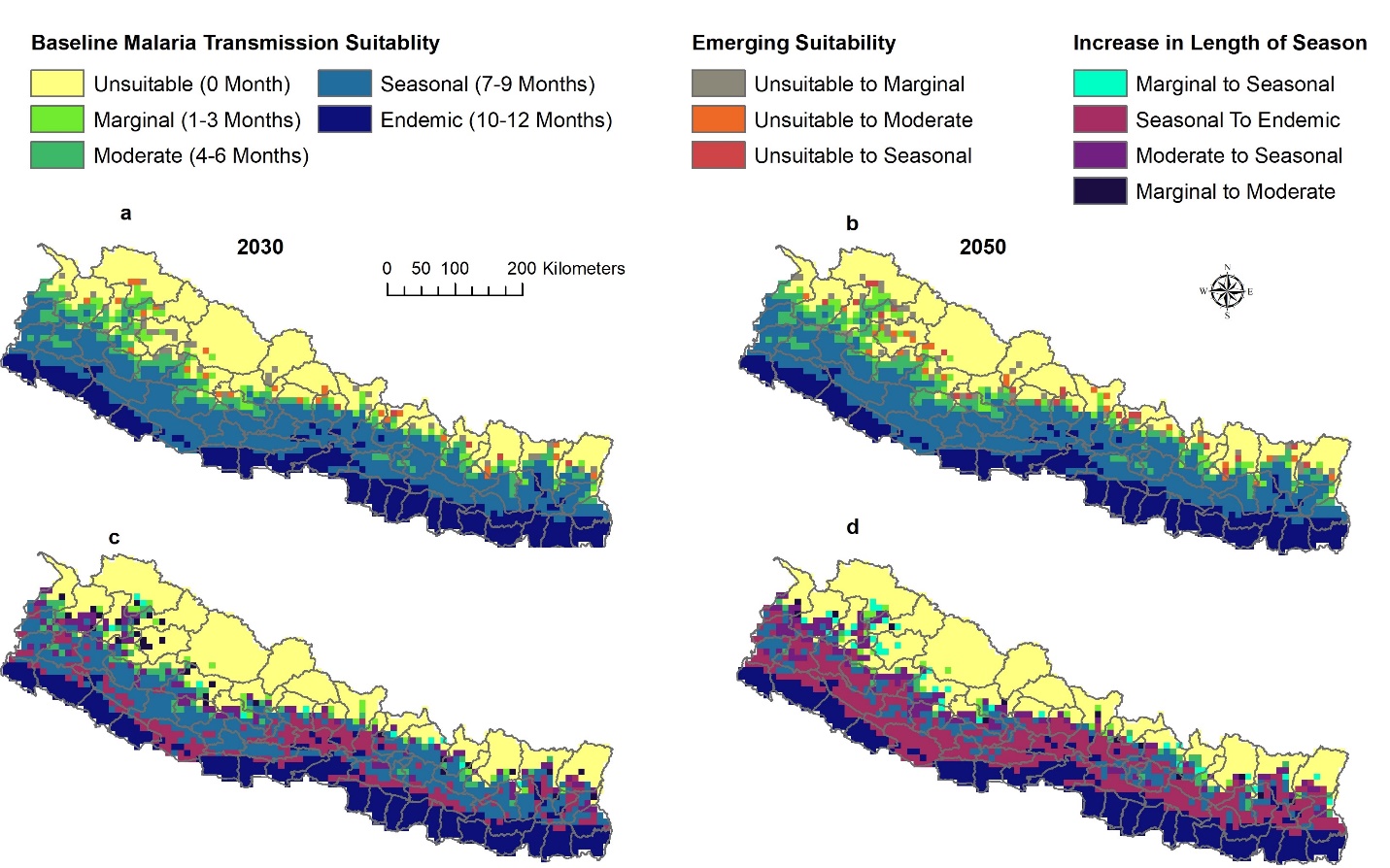


**Fig. S7: Shifting malaria transmission suitability by ASPV under RCP 4.5.** Areas with Emerging Suitability (a-b) and Areas with Increase in Length of Season (c-d).


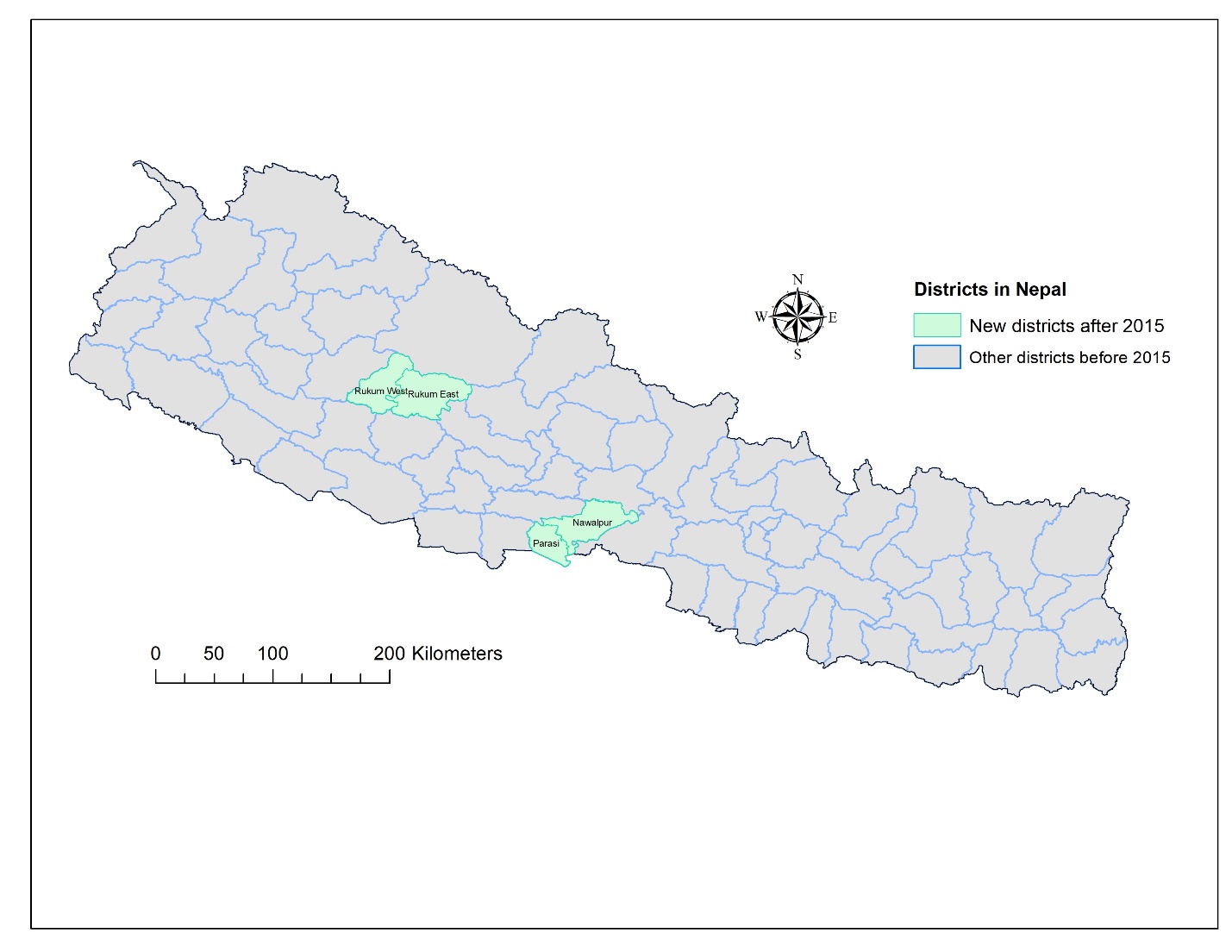


**Fig. S8: Newly formed districts in Nepal after 2015.**

**Table S1: Warren’s I similarity metrics for comparing malaria transmission suitability between ASPF and ASPV**

| **Comparison layers** | **Warren’s I metric** |
| --- | --- |
| *Warren’s I similarity metric between ASPF and ASPV* | |
| ASPF vs ASPV for RCP 4.5 2030 | 0.999 |
| ASPF vs ASPV for RCP 4.5 2050 | 0.999 |
| ASPF vs ASPV for RCP 8.5 2030 | 0.999 |
| ASPF vs ASPV for RCP 8.5 2050 | 0.999 |
| *Warren’s I similarity metric between two RCPs for ASPF* | |
| RCP 4.5 vs RCP 8.5 for 2030 | 0.999 |
| RCP 4.5 vs RCP 8.5 for 2050 | 0.998 |
| 2030 vs 2050 for RCP 4.5 | 0.998 |
| 2030 vs 2050 for RCP 8.5 | 0.999 |
| *Warren’s I similarity metric between two RCPs for ASPV* | |
| RCP 4.5 vs RCP 8.5 for 2030 | 0.999 |
| RCP 4.5 vs RCP 8.5 for 2050 | 0.999 |
| 2030 vs 2050 for RCP 4.5 | 0.998 |
| 2030 vs 2050 for RCP 8.5 | 0.998 |

**Table S2:** Differences between malaria transmission suitability by ASPF and ASPV.

| Change in Malaria Transmission Suitability | RCP 4.5 | | | | RCP 8.5 | | | |
| --- | --- | --- | --- | --- | --- | --- | --- | --- |
|  | 2030 | | 2050 | | 2030 | | 2050 | |
|  | ASPF | ASPV | ASPF | ASPV | ASPF | ASPV | ASPF | ASPV |
| Moderate to Endemic | No | Yes | Yes | Yes | No | No | Yes | Yes |
| Moderate to Unsuitable | Yes | Yes | Yes | No | Yes | Yes | Yes | No |
| Endemic to Seasonal | Yes | No | No | Yes | Yes | Yes | No | Yes |

**Note:** Yes means the conversion in category will occur and No means the conversion in category will not occur.
